# A bibliometric approach to worldwide scientific production of familial hypophosphataemic rickets in Scopus (2000–2022)

**DOI:** 10.1186/s13023-025-04105-4

**Published:** 2025-11-18

**Authors:** Frank Hernández-García, Helena Gil-Peña, José M. López, Rocío Fuente, Patricia Oro Carbajosa, Ibraín Enrique Corrales-Reyes, Julián Rodríguez Suárez

**Affiliations:** 1 Departmento de Medicina, Facultad de Medicina, Universidad de Oviedo, Asturias, Spain; 2 AGC de Pediatría, Hospital Universitario Central de Asturias, Asturias, Spain; 3 Departamento de Morfología y Biología Funcional, Facultad de Medicina, Universidad de Oviedo, Asturias, Spain; 4 Departamento de Enfermería, Facultad de Ciencias Biomédicas y de la Salud, Universidad Europea de Madrid, Madrid, Spain; 5 Sarasota Oral and Implant Surgery, Sarasota, Florida USA; 6 Grupo de Investigación en Pediatría, Instituto de Investigación Sanitaria del Principado de Asturias, Asturias, Spain; 7 Grupo de Investigación GRUPIN, Uniovi-Pediatría OviPed, Universidad de Oviedo, Asturias, Spain

**Keywords:** Bibliometrics, Familial hypophosphatemic rickets, Rickets, FGF23 protein, PHEX protein, X-linked hypophosphatemic rickets, Burosumab

## Abstract

**Background:**

Familial hypophosphatemic rickets are disabling conditions that negatively impact physical functioning, activities of daily living, mental health and social life. The most common cause of hypophosphatemic rickets is genetic factors, such as X-linked hypophosphatemia. The evaluation of the scientific application of familial hypophosphataemic rickets aids in understanding the research landscape, identifying opportunities for improvement, and promoting significant advancements in the understanding and treatment of this medical condition. This study aimed to characterize the worldwide scientific production of articles on familial hypophosphatemic rickets indexed in Scopus, through the analysis of publication growth rate, leading countries and journals, international collaboration networks, and predominant research keywords.

**Methods:**

An observational, descriptive, and cross-sectional study was conducted through a bibliometric analysis of the worldwide scientific production of familial hypophosphatemic rickets published in journals indexed in Scopus from 2000 to 2022. To retrieve the publications, Scopus was accessed on April 4, 2023, and an advanced search was performed using a filter by title, abstract and key words, source (journals), publication year, and type of article (article and review). The search terms used were extracted from the PubMed Medical Subject Headings (MeSH) related to the disease included in the MeSH catalog. Additionally, an analysis of co-occurrence between countries and keywords was carried out with VOSviewer software.

**Results:**

This study identified 1,269 articles on familial hypophosphatemic rickets (938 articles and 331 reviews). In total, 39,548 citations were received, with an H index of 95. The majority of the articles (76.9%) were published in high-impact journals according to quartile (Q), Q1 and Q2. Scientific production has shown a growing trend in recent years. The countries with the highest scientific production are the U.S., Japan, and the United Kingdom, considering that low- and middle-income countries contribute less to international scientific production. Among 1,858 author keywords, 109 met the inclusion threshold (≥ 6 occurrences) and were grouped into five thematic clusters related to genetics, treatment, pathophysiology, complications, and clinical manifestations. Recent research trends highlight increasing focus on burosumab, quality of life, and chronic complications in familial hypophosphatemic rickets.

**Conclusions:**

Scientific production has shown sustained growth in recent years. The U.S. solidifies itself as the country leading scientific production on familial hypophosphatemic rickets.

**Supplementary Information:**

The online version contains supplementary material available at 10.1186/s13023-025-04105-4.

## Graphical Abstract

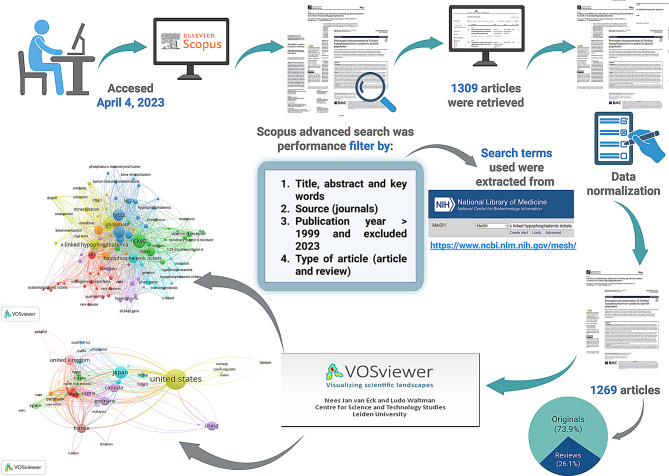

## Background

Familial hypophosphatemic rickets are a group of genetic disorders characterized by renal phosphate wasting, resulting in hypophosphatemia, rickets, and normal serum calcium levels. The clinical features of these patients include short stature, bone pain, and skeletal deformities. Hypophosphatemic rickets typically present in infancy or early childhood with skeletal deformities in the early stages of life [1]. The most common cause of hypophosphatemic rickets is genetic, such as X-linked hypophosphatemia (XLH) [2].

An estimated prevalence of less than 1 case per 100,000 people has been reported for autosomal dominant hypophosphatemic rickets and hypophosphatemic rickets with hypercalciuria [3, 4]. In some regions, the estimated incidence reaches 2.03 cases per 100,000 inhabitants [5]. The worldwide incidence of XLH is reported to be 1 to 9 cases per 100,000 habitants [6]. According to other studies, the estimated incidence of XLH is 14.0 (95% CI: 10.8–18.1) per million people [7].

Familial hypophosphatemic rickets have been described as disabling conditions for those affected, with a negative impact on physical functioning, activities of daily living, mental health, social life, and leisure activities. In adulthood, it is associated with a substantial disease burden and decreased quality of life, where individuals often experience severe pain and progressive disability [8–11].

Bibliometrics is the application of mathematics and statistical methods to any written source on the basis of communication facets and considers elements such as authors, publication title, document type, language, abstract, and keywords or descriptors [12, 13]. Bibliometric studies, in any branch of science, represent a valuable tool in the era of information and communications. They serve not only as instruments for evaluating the scientific production of a particular subject but also to enhance and increase the excellence of scientific research to higher levels [13].

Research in the health sciences serves as the cornerstone upon which health services develop and progress, refining medical care at various levels. Evaluating scientific production on a specific disease or health issue is strategically important for health policy decision-making at the national or regional level. This provides a general overview of the value assigned to a topic by the scientific community, based on the research conducted on it. The evaluation of the worldwide scientific production of familial hypophosphataemic rickets can aid in understanding the research landscape, identifying opportunities for improvement, and promoting significant advancements in the understanding and treatment of this medical condition.

As far as has been reviewed, no studies evaluating the worldwide scientific production of familial hypophosphatemic rickets have been found in the main databases for scientific information retrieval. This study aims to characterize the worldwide scientific production of articles on familial hypophosphatemic rickets indexed in Scopus, through the analysis of publication growth rate, leading countries and journals, international collaboration networks, and predominant research keywords.

## Methods

### Design

An observational, descriptive, and cross-sectional study was conducted through a bibliometric analysis of the worldwide scientific production of familial hypophosphatemic rickets published in journals indexed in Scopus between 2000 and 2022. This study period was chosen because of the notably limited number of earlier publications identified in an initial search, which precluded a thorough examination. As such, this period serves as a representative sample of the global scientific literature on familial hypophosphatemic rickets.

The analysis included only original research articles and review papers. Letters to the editor and conference proceedings were excluded, as these types of documents generally lack adequate bibliometric data and often have not been subjected to peer review.

### Bibliometric indicators

The following bibliometric indicators were studied:

- Number of documents (Ndoc).
- Articles in English (Ndoc Eng). Articles published in English.
- Non-English article (Ndoc Non-Eng). Articles published in a language other than English.
- Overlap (Ndoc Overlap). Articles published in two languages.
- Citations (NCit). Total citations were obtained from articles indexed in Scopus.
- Cited articles (Cited doc). Total number of published articles that have been cited at least once according to Scopus.
- Citations per document (Cpd). Average number of received citations.
- H-index. This index considers both the number of articles and the citations they receive. An author has an h = x index if he/she has x articles that have been cited at least x times [14]. This indicator is also used to characterize groups (a group of authors, a department, or a country).
- R-index: Square root of the total number of citations received by the core articles contributing to the h-index [15].
- Growth rate (GR): percent change in the number of articles published in a domain with respect to the previous year, calculated as follows: GRn = [(Ndocn − Ndocn − 1)/Ndocn − 1] * 100, where **n** is the year [16].
- Quartiles (Q). According to the SCImago Journal & Country Rank (SJR), the journals indexed in Scopus are placed in quartiles, where those in the first quartile have the highest impact. There are journals that do not appear in the ranking (nonranked) because of their recent inclusion in the database.
- High-quality publications (Ndoc Q1). Percentage of publications in journals included in the quartile of maximum visibility.
- Scientific leadership (% Lead). Percentage of articles from a country in which the corresponding author belongs to a national institution. These are referred to as lead documents [17].
- % Q1 lead (Ndoc Q1 lead). The percentage of articles in journals included in the first quartile in which the corresponding author is affiliated with an institution who belongs to the same country [18].

### Data collection and search strategy

To retrieve the publications, Scopus (Elsevier BV Company, Netherlands, http://www.scopus.com*)* was the database chosen to identify the studies because it is considered the most complete database worldwide, encompassing 100% of the publications indexed in MEDLINE. Scopus was accessed on April 4, 2023, and an advanced search was performed using a filter by title, abstract and key words, source (journals), publication year, and type of article (article and review). The search terms used were extracted from the PubMed Medical Subject Headings (MeSH) related to the disease included in the MeSH catalog. The search strategy is shown in Supplementary Material 1.

Visualization map analysis according to the authors’ keywords was performed via VOSviewer version 1.6.19 to analyze the co-occurrence of the collaborating countries and keywords via the full-counting method [19, 20]. The graphical interpretation is based on the grouping guidelines (keywords) and the distance between countries (co-occurrence networks).

Additionally, descriptive statistics were performed via Microsoft Excel 2019^®^, which calculates the absolute and relative frequencies for each variable of the study.

### Ethics

The data were downloaded from public databases; therefore, no ethical approval was necessary. The final database, although derived from public domain information, was standardized and developed by the authors.

## Results

### General bibliometric indicators

Initially, 1309 articles were retrieved, and after normalization, 1269 were included. For the Scopus indices, 1269 articles received 39,548 citations, with an average of 31.2 citations per document (Table 1). The ratio of original articles to reviews was 2.8. The overall values of the H and R indices are 95 and 143.2, respectively. Additionally, 86% of the publications were cited (*n* = 1091); 1006 had 1-100 citations; 56 had 101–200 citations; 14 had 201–300 citations; four had 301–400 citations; five had 401–500 citations; and the remaining six had more than 500 citations. The most cited article has received 1284 citations and was published in *Nature Genetics*, which belongs to the first quartile of visibility in Scopus titled “Autosomal dominant hypophosphataemic rickets is associated with mutations in FGF23”, by Kenneth E. White and Bettina Lorenz-Depiereux et al. (Supplementary Material 2).

**Table 1:** General bibliometric indicators of scientific production worldwide

Indicator	Value
Ndoc	1269
Ndoc Eng (%)	1158 (91.3)
Ndoc Non-Eng (%)	96 (7.7)
Ndoc Overlap (%)	15 (1.2)
Articles (%)	938 (73.9)
Reviews (%)	331 (26.1)
NCit	39,548
Cited doc (%)	1091 (86.0)
Cpd	31.2
H index	95
R index	143.2

More than half of the articles (56.6%; *n* = 718) were published in high-visibility journals (Q1), all in the English language, with 70.9% of them corresponding to original articles. Among these, 675 documents were cited, with an average H-index of 91 (Table 2).

**Table 2:** Bibliometric indicators according to quartiles of visibility

Indicators	Q1	Q2	Q3	Q4	Non ranked
Ndoc (%)	718 (56.6)	257 (20.3)	117 (9.2)	79 (6.2)	98 (7.7)
Ndoc Eng (%)	718 (100)	253 (98.4)	96 (82.1)	36 (45.6)	55 (56.1)
Ndoc Non-Eng (%)	0	2 (0.8)	11 (9.4)	41 (51.9)	42 (42.9)
Ndoc Overlap (%)	0	2 (0.8)	10 (8.5)	2 (2.5)	1 (1.0)
Articles (%)	538 (74.9)	204 (79.4)	83 (70.9)	59 (74.7)	54 (55.1)
Reviews (%)	180 (25.1)	53 (20.6)	34 (29.1)	20 (25.3)	44 (44.9)
NCit	33,157	3764	949	244	1434
Cpd	46.2	14.6	11.1	3.1	14.6
Cited doc (%)	675 (94.0)	215 (83.7)	93 (79.5)	38 (48.1)	70 (71.4)
H index	91	34	17	7	17
R index	140.1	44.8	24.4	13.2	34.6

Supplementary Material 3 displays scientific production over the years, along with corresponding bibliometric indicators.

### Bibliometric indicators of production and impact on journals

The bibliometric indicators and impacts on journals with ≥ 15 papers are shown in Table 3. The *Journal of Bone and Mineral Research* is the most frequently chosen journal by researchers for publication. Its 67 articles have received 4507 citations and have an H-index of 31. In terms of impact, as evaluated through the citations received, the articles published in this journal had the highest results, reaching a high average number of citations per paper (67.3).

**Table 3:**
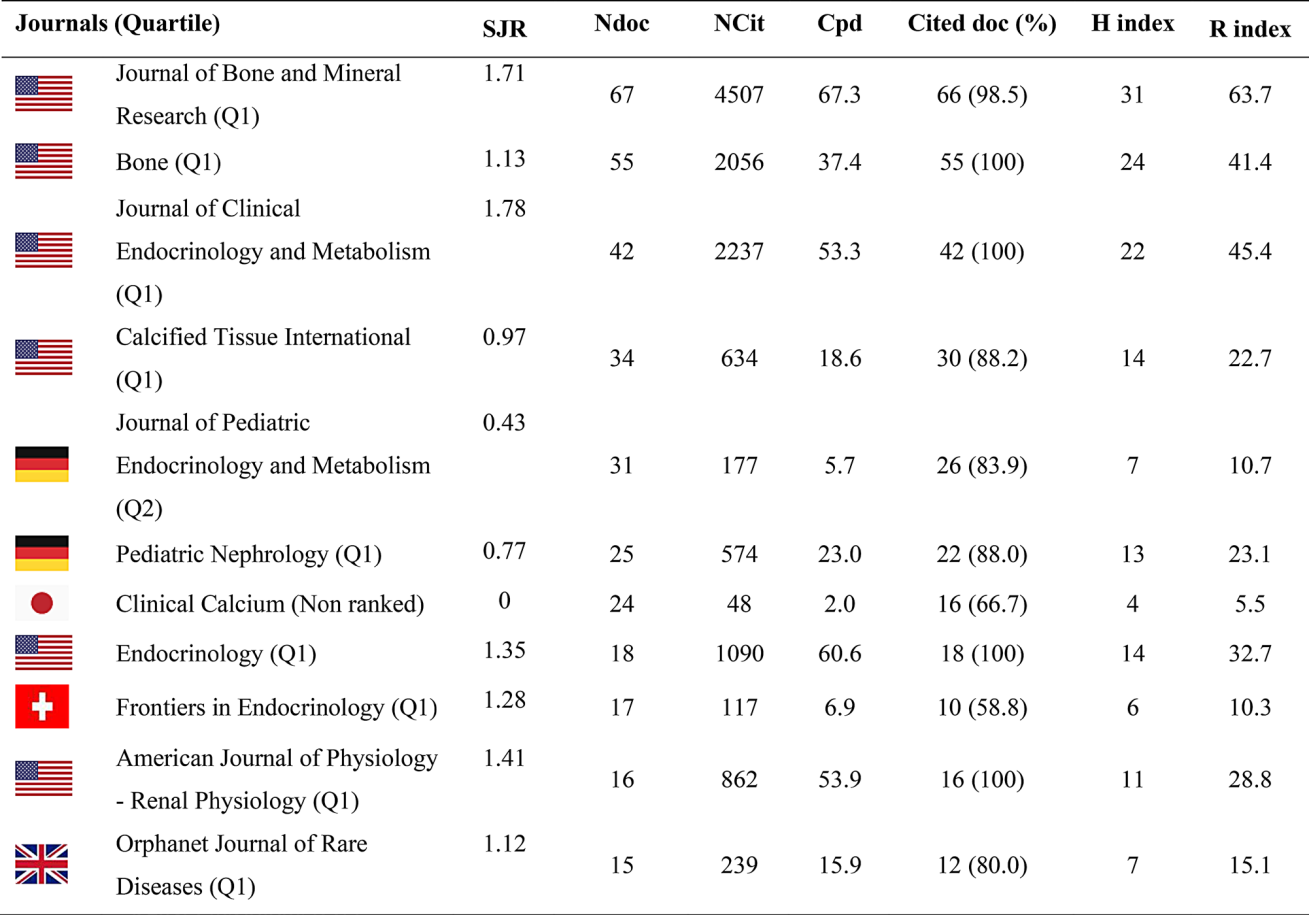
Bibliometric indicators of production and impact on journals with fifteen or more articles

**Table 4:**
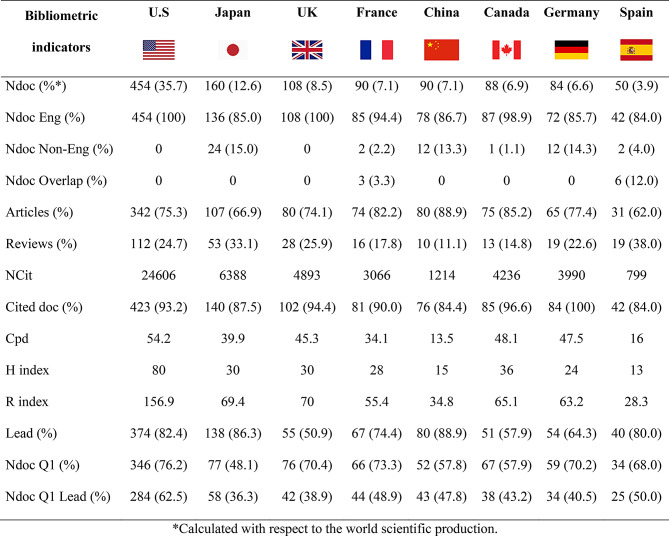
Bibliometric indicators of the production, impact, and scientific leadership of countries with fifty or more articles

### Scientific leadership

Table 4 shows the distribution of scientific production by country with fifty or more articles, which shows a predominance of documents from the U.S. (*n* = 454), representing 35.7% of the total number of published articles. In terms of impact according to the number of citations received, this figure far surpasses that of the other countries since its articles have received 24,606 citations, with a high average number of citations per document and H and R indices (Cpd = 54.2; H index = 80; R index = 156.9). Japan is the country with the second highest scientific production since its 160 articles have received 6388 citations (Cpd = 69.29; H index = 42; R index = 412.94).

A total of 82.4% of the articles from the U.S. have a corresponding author affiliated with a national institution. Among these articles, 62.5% were published in first quartile journals.

The most productive institutions were Indiana University School of Medicine (*n* = 64), Université McGill (*n* = 50), Yale School of Medicine (*n* = 50), AP-HP Assistance Publique - Hopitaux de Paris (*n* = 49), Université Paris Cité (*n* = 41), Inserm (*n* = 40) and Harvard Medical School (*n* = 40).

**Fig. 1:**
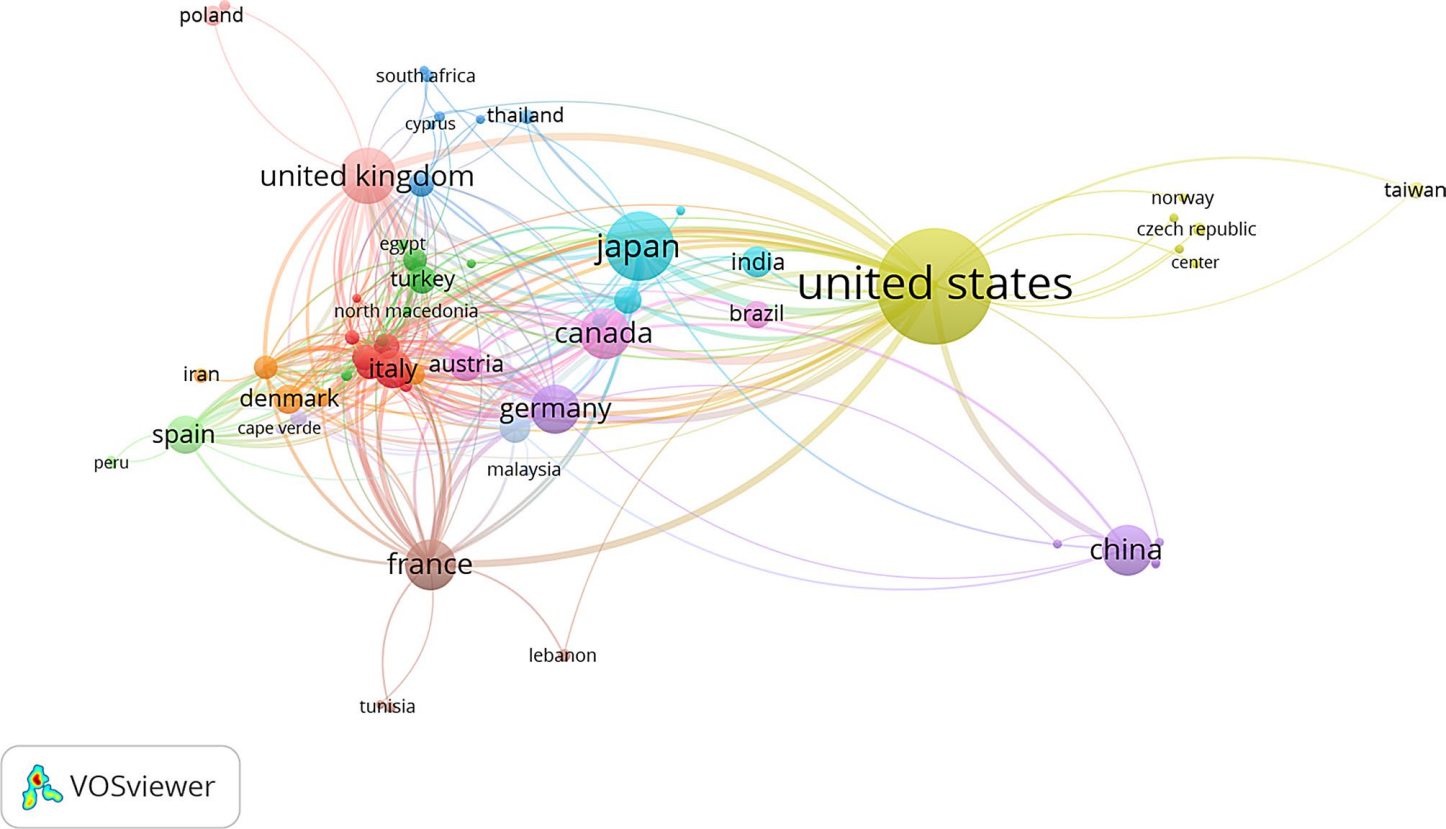
Scientific collaboration networks among countries with ≥ 5 articles published (Scopus, 2000–2022)

There is extensive international scientific collaboration led by researchers in the U.S., Japan and Europe (Fig. 1). The low- and middle-income countries, fundamentally in Africa and Latin America, are the least represented and productive in relation to research on familial hypophosphatemic rickets.

### Research trends

**Fig. 2:**
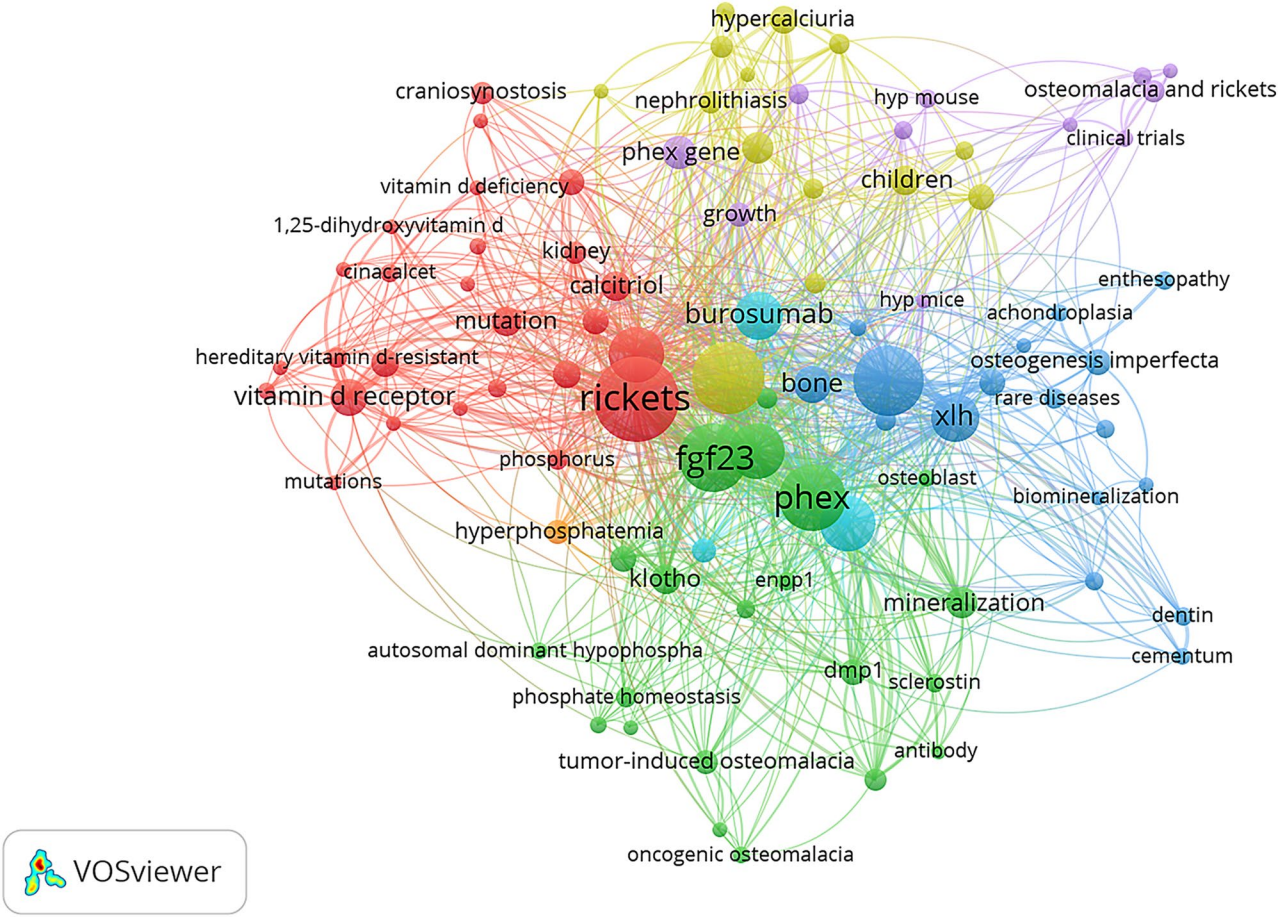
Author keywords and research topics. The map is based on text data from the scientific production of XLH (2000–2022)

Among the 1858 authors’ keywords, 109 were selected for analysis in VOSviewer and were chosen on the basis of their occurrence in at least 6 documents (Fig. 2). The keywords most frequently used by the authors were rickets (*n* = 188), FGF23 (*n* = 123), hypophosphataemia (*n* = 137), PHEX (*n* = 117), phosphate (*n* = 84), vitamin D (*n* = 82), osteomalacia (*n* = 81), X-linked hypophosphatemic rickets (*n* = 74) and burosumab (*n* = 60).

The 109 keywords were divided into five different groups according to the following terms: (1) “genetic implications” (PHEX, mutation, Hyp mice/Hyp mouse); (2) “management and treatment” (burosumab, phosphate, cinacalcet); (3) “pathophysiology relationships and protein related” (Dmp-1, osteopontin, sclerostin, 1,25 dihydroxyvitamin D, phosphate metabolism); (4) “complication and quality of life” (nephrocalcinosis, hyperparathyroidism); and (5) “clinical and analytical manifestations relationship” (hypophosphatemia, Rickets, Osteomalacia, phosphaturia).

**Fig. 3:**
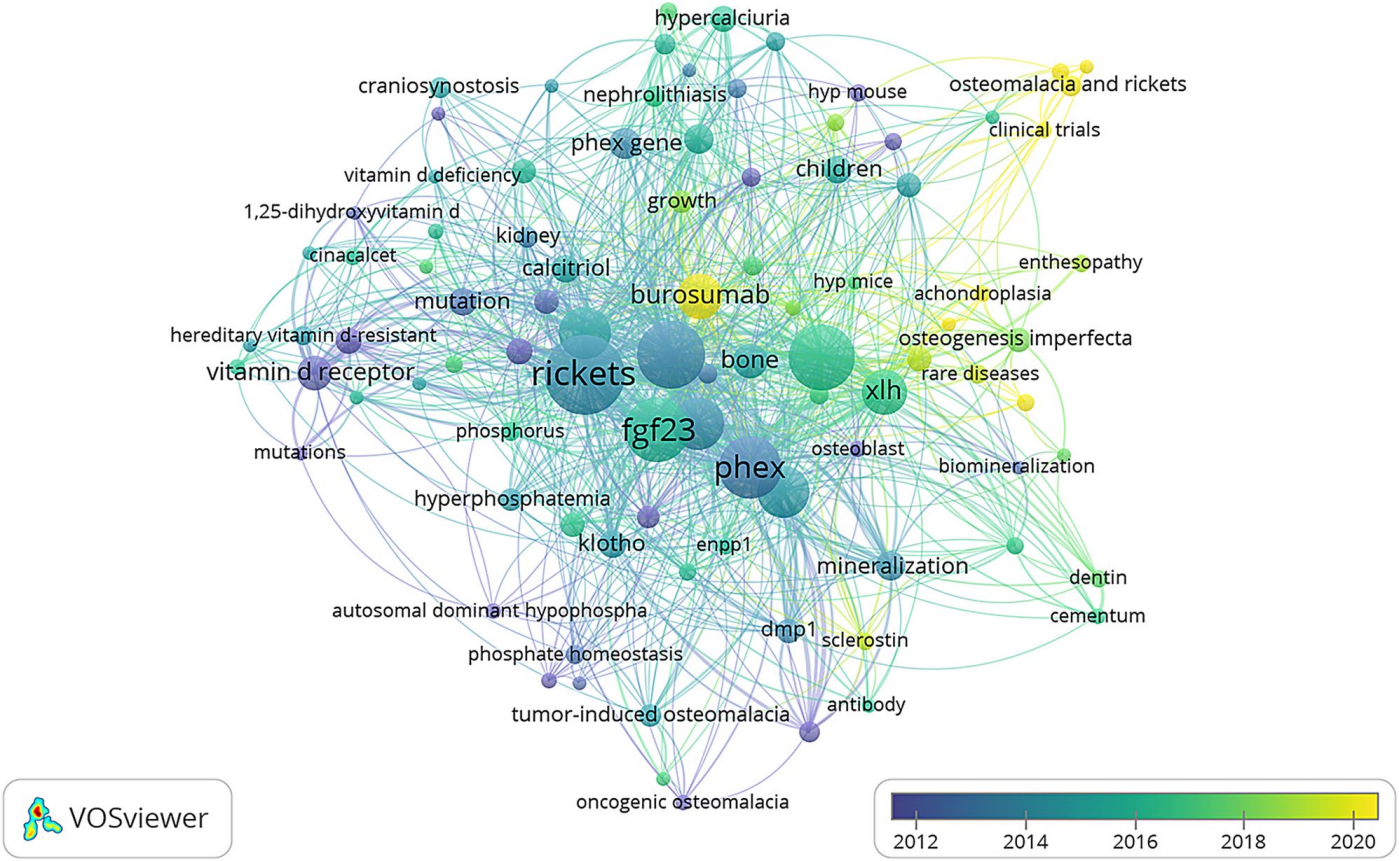
Annual visualization of author's keywords and research topics. The map is based on text data from the scientific production of XLH (2000–2022)

Figure 3 shows the evolution of keywords and research trends in familial hypophosphatemic rickets over the years. In the last 5 years, research on burosumab, quality of life and chronic complications of this disease has gained prominence, becoming a trend in the investigation of familial hypophosphatemic rickets and XLH.

## Discussion

The present research constitutes the first study analyzing the individual scientific basis of a rare disease, specifically familial hypophosphatemic rickets. The selection of Scopus was based on its comprehensive coverage, which facilitates the inclusion of journals across all quartiles (ranging from higher to lower impact), alongside publications not indexed by more restrictive databases, namely, MEDLINE or Web of Science (WoS).

The scientific landscape of rare diseases has rapidly evolved, with expectations for this change to accelerate. Individuals living with rare diseases increasingly benefit from new therapies, and related research has been conducted. By definition, rare diseases are uncommon, leading to fewer patients, physicians, and researchers dedicated to them, as is the case with familial hypophosphataemic rickets. The infrastructure required to conduct studies in this area can at times become a costly endeavor, especially when considering per-participant research expenses.

In comparison with other medical conditions, scientific production on familial hypophosphatemic rickets could be considered rare, on the basis of the finding of only 1269 indexed documents over 22 years. Given its status as a group of rare and low-prevalence diseases, scientific activity focused on this disease is reserved for specialized research groups and reference institutions in the field. The prevalence of other diseases, whose diagnosis is centered around the pediatric age, is greater than that of other diseases [21–24]; however, in some of these studies, the evaluated period was longer than that of our study. Escudero Gómez et al. [25] also reported low scientific production of rare diseases in Spain, where they retrieved only 2,978 documents when analyzing studies involving three types of rare diseases: congenital anomalies, inborn errors of metabolism, and primary immunodeficiencies. In the specific field of bone defects, other authors [26] have also focused on the slow growth of scientific production in this area, although it has maintained an increasing trend.

In our study, a high percentage of published articles (86%) were found, with an average of 31.2 citations per document and a high H-index. The high citation rate of these studies could be attributed primarily to the limited available literature on rare diseases, which results in less dispersion of the most relevant studies across the scientific literature. Furthermore, the fact that most studies are published in high-impact Q1 and Q2 journals favors this aspect, as these journals are known to have greater visibility.

The most productive publications on familial hypophosphatemic rickets are expected to be found in the SJR thematic area corresponding to Endocrinology, Diabetes, and Metabolism. *Journal of Bone and Mineral Research*, *Bone* and *Journal of Clinical Endocrinology and Metabolism* are the most productive journals in this field, with the highest number of documents and citations and the highest H-index. Conversely, other studies have noted *Bone* and *Journal of Bone and Mineral Research* as highly prolific in bone defect research, which contrasts with our findings where they were not the principal publications [26]. Another author also found the *Journal of Clinical Endocrinology and Metabolism* to be one of the most cited journals on the scientific productivity of Spanish pediatrics [27]. A study conducted by Chhapola V. et al. [28], where they analyzed citations of classic pediatric studies, revealed that the pediatric subspecialties of growth and development and endocrinology were the most cited and productive.

The growth rate of scientific production has fluctuated since 2000, with both positive and negative values. However, since 2017, it has remained consistently positive, reaching its peak value in 2022 at 11.2. Notably, before 2020, no more than 100 publications on familial hypophosphatemic rickets were indexed in the database. In recent years, this figure has surpassed 100 points. Therefore, in comparison with other thematic areas, scientific production in familial hypophosphatemic rickets is relatively limited. Importantly, these conditions have low incidence and prevalence rates and are sometimes underdiagnosed [5, 29].

Globally, in correspondence with other research areas [21–23, 28, 30], the U.S. is the most productive country in terms of familial hypophosphatemic rickets, contributing to more than a quarter of the world’s scientific production. Kong et al. [31] and Tian M et al. [32] similarly reported that the U.S. is the top producer of rare diseases. European countries were also among the primary producers, with Spain notably ranking within the top ten. These studies, however, did not restrict their scope to a single pathology, and their search encompassed WoS and the China National Knowledge Infrastructure (CNKI). This finding is consistent with our own research by Haixiong Lin et al. [26], which identified the U.S. as leading the trend in bone defect research, followed by China, Japan, and European Union countries.

This fact may be attributed, among other factors, to the presence of some of the world’s leading medical research centers and specialized hospitals for genetic diseases in the U.S. Significant investment in research and development in medical and scientific fields by government agencies, nonprofit organizations, and the private industry contributes to research leadership. These investments enable the conduct of studies, clinical trials, and the development of innovative treatments. The advanced technological infrastructure in the U.S., which includes genetic sequencing facilities, bioinformatics, and other cutting-edge technologies, allows researchers to conduct detailed studies on genetic diseases and develop more effective therapeutic approaches. The quality of education and the ability to attract and retain highly trained professionals in scientific and medical fields are crucial factors for research leadership.

International collaboration networks are crucial in medical research. Research collaboration can help increase scientific productivity [33]. The U.S. leads in terms of its broad collaboration network, followed by Japan, which is the second most productive country. Third, the European region, led by the United Kingdom, France, Germany, and Spain, could be attributed. Several of the primary research groups are in these countries. These results align with the findings of other authors, where the U.S. and Europe led scientific collaboration [21, 22]. Low-income countries, primarily in Africa and some in Latin America, have the lowest scientific production of XLH, and in some cases, it is nonexistent. Several bibliometric studies in the field of pediatrics have shown low indexed scientific production in these regions [22, 23, 30].

The International Rare Disease Research Consortium (IRDiRC) has established a promising initiative in rare disease research; nevertheless, much work still needs to be done in this field. This is especially pertinent given that less than 6% of rare diseases have approved treatments, and the majority of drug development efforts are concentrated on a limited number of diseases [19]. Precise data on the quantification and progress of XLH are not currently available. The International X-Linked Hypophosphatemia (XLH) Registry provides an approach to these data [29]. Other projects and research groups, both basic and clinical, have focused on the study of familial hypophosphatemic rickets and other rare diseases, with primary tubulopathy as the physio pathological basis [34]. There is a need to develop a common high-quality platform for registering rare bone and mineral conditions [35].

The analysis of keywords revealed several significant trends in research on X-linked hypophosphatemic rickets (XLH). The most frequent keywords provide a comprehensive view of the focus areas in the scientific literature. The generic name of the disease, “Rickets,” and “X-linked hypophosphatemic rickets” are key terms, emphasizing the importance of understanding the disease from both clinical and genetic perspectives. Terms such as “FGF23,” “PHEX,” and “phosphate” suggest a focus on understanding the underlying pathophysiological and genetic mechanisms of XLH. The presence of “osteomalacia” as a keyword indicates an emphasis on investigating complications associated with the disease, delving into long-term effects on bone tissue. With the introduction of burosumab as a new therapy for XLH, research on this topic has influenced its positioning as a keyword used by authors in both basic research and clinical trials. The inclusion of “burosumab” and “vitamin D” reflects the interest in therapeutically addressing XLH, with special attention given to the new therapy burosumab. The use of burosumab as a keyword has a significant impact on related research, both in basic studies and in clinical trials. Figure 3 shows that burosumab appears to be a central research focus in the study of XLH.

The focus on “chronic complications” suggested a shift toward understanding the long-term effects of the disease. Additionally, the study of “quality of life” reflects a growing interest in addressing broader aspects of patient well-being. The evolution of keywords reflects not only progress in the scientific and therapeutic understanding of familial hypophosphatemic rickets but also a shift toward more holistic considerations that encompass the quality of life and chronic complications of patients.

There was no apparent association between color and the research theme, which may be due to the overlap of these terms across different articles.

Some limitations of our study should be addressed in future scientometric research. Despite selecting Scopus for its recognition as the most comprehensive database, several studies published in non-indexed journals, those in the process of indexing, or preprint repositories may have been excluded from the analysis. Additionally, many studies from low-income countries tend to be published in local journals that are not indexed, potentially affecting their perceived productivity in the context of hypophosphatemic rickets. The inclusion of additional databases, such as Web of Science, MEDLINE, Dimensions, and SciELO, would have provided broader and more comprehensive coverage of the scientific literature. On a different note, a further limitation to address in subsequent research is the consideration of altmetrics, or the social impact of investigations, as a study variable, which is consistent with the rising trend of leveraging social networks for scientific communication.

Moreover, owing to constantly changing citation volumes over time, the results of our study are temporary and valid. Nevertheless, we believe that our study provides a detailed international scientometric analysis of the scientific production of familial hypophosphatemic rickets.

The results suggest the need to promote international cooperation through engagement with leading research groups and institutions.

## Supplementary Information

Below is the link to the electronic supplementary material.

Supplementary Material 1

Supplementary Material 2

Supplementary Material 3

## Data Availability

The datasets generated and analyzed during the current study are available in the Harvard Dataverse repository 10.7910/DVN/X0NUZY.
