# Supplementary material for: A bibliometric approach to worldwide scientific production of familial hypophosphataemic rickets in Scopus (2000–2022)
Source: Orphanet J Rare Dis. 2025 Nov 18;20:589. doi: 10.1186/s13023-025-04105-4 (PMC12625217; doi:10.1186/s13023-025-04105-4)
Supplement: Supplementary file 1 — Supplementary Material 1 [file 13023_2025_4105_MOESM1_ESM.docx]

**Supplementary Material 1. Search strategy.**

| **Operator** | **Field** | **Search term** |
| --- | --- | --- |
|  | TITLE-ABS-KEY | "x linked hypophosphatemic" OR "x-linked hypophosphatemic" OR "Generalized Resistance To 1,25 Dihydroxyvitamin D" OR "Generalized Resistance To 1,25-Dihydroxyvitamin D" OR "Hereditary Hypophosphatemic Rickets" OR "Hereditary Vitamin D Resistant Rickets" OR "Hereditary Vitamin D-Resistant Rickets" OR "Hypocalcemic Vitamin D Resistant Rickets" OR "Hypocalcemic Vitamin D-Resistant Rickets" OR "X Linked Hypophosphatemia" OR "X-Linked Hypophosphatemia" OR "Familial Hypophosphatemic Rickets" OR "Hereditary Hypophosphatemic Rickets" OR "X Linked Dominant Hypophosphatemic Rickets" OR "X Linked Recessive Hypophosphatemic Rickets" OR "X-Linked Recessive Hypophosphatemic Rickets" OR "X-Linked Dominant Hypophosphatemic Rickets" OR "Familial Hypophosphatemic Rickets" OR "Hereditary Hypophosphatemic Rickets" OR "Hereditary Vitamin D Resistant Rickets" OR "Hereditary Vitamin D-Resistant Rickets" OR "X-Linked Hypophosphatemic Rickets" OR "Vitamin D Resistant Rickets With End Organ Unresponsiveness To 1,25 Dihydroxycholecalciferol" OR "Hereditary Vitamin D Resistant Rickets" OR "X Linked Vitamin D Resistant Rickets" OR "X-Linked Vitamin D Resistant Rickets" OR "Vitamin D-Resistant Rickets With End-Organ Unresponsiveness To 1,25-Dihydroxycholecalciferol" OR "Hereditary Vitamin D-Resistant Rickets" OR "X-Linked Vitamin D-Resistant Rickets" OR "X Linked Hypophosphatemia" OR "X-Linked Hypophosphatemia" OR "X-Linked Hypophosphatemic Rickets" |
| AND | SRCTYPE | j |
| AND | PUBYEAR | > 1999 |
| AND | LIMIT-TO | DOCTYPE, "ar" OR DOCTYPE, "re" |
| AND | EXCLUDE | PUBYEAR , 2023 |
