# Supplementary material for: A bibliometric approach to worldwide scientific production of familial hypophosphataemic rickets in Scopus (2000–2022)
Source: Orphanet J Rare Dis. 2025 Nov 18;20:589. doi: 10.1186/s13023-025-04105-4 (PMC12625217; doi:10.1186/s13023-025-04105-4)
Supplement: Supplementary file 2 — Supplementary Material 2 [file 13023_2025_4105_MOESM2_ESM.docx]

**Supplementary Material 2. The most cited articles.**

| **Authors** | **Title** | **Year** | **Publication** | **Cite** | **Correspondence author** |
| --- | --- | --- | --- | --- | --- |
| White K.E., Evans W.E., O'Riordan J.L.H., Speer M.C., Econs M.J., Lorenz-Depiereux B., Grabowski M., Meitinger T., Strom T.M. | Autosomal dominant hypophosphataemic rickets is associated with mutations in FGF23 | 2000 | Nature Genetics (Q1) | 1284 | Department of Medicine, Indianapolis, IN, United States |
| Christakos S., Dhawan P., Verstuyf A., Verlinden L., Carmeliet G. | Vitamin D: Metabolism, molecular mechanism of action, and pleiotropic effects | 2015 | Physiological Reviews (Q1) | 983 | Department of Microbiology, Biochemistry and Molecular Genetics, Rutgers, The State University of New Jersey, New Jersey Medical School, Newark, New Jersey; and Laboratory of Clinical and Experimental Endocrinology, KU Leuven, Leuven, Belgium |
| Shane E., Burr D., Ebeling P.R., Abrahamsen B., Adler R.A., Brown T.D., Cheung A.M., Cosman F., Curtis J.R., Dell R., Dempster D., Einhorn T.A., Genant H.K., Geusens P., Klaushofer K., Koval K., Lane J.M., McKiernan F., McKinney R., Ng A., Nieves J., O'Keefe R., Papapoulos S., Sen H.T., Van Der Meulen M.C., Weinstein R.S., Whyte M. | Atypical subtrochanteric and diaphyseal femoral fractures: Report of a task force of the american society for bone and mineral Research | 2010 | Journal of Bone and Mineral Research (Q1) | 903 | Columbia University, College of Physicians and Surgeons, PH 8 West 864, 630 West 168th Street, New York, NY 10032, USA |
| Jonsson K.B., Zahradnik R., Larsson T., White K.E., Sugimoto T., Imanishi Y., Yamamoto T., Hampson G., Koshiyama H., Ljunggren Ö., Oba K., Yang I.M., Miyauchi A., Econs M.J., Lavigne J., Jüppner H. | Fibroblast growth factor 23 in oncogenic osteomalacia and X-linked hypophosphatemia | 2003 | New England Journal of Medicine (Q1) | 769 | Endocrine Unit, , Boston, MA 02114, United States; |
| Yamazaki Y., Okazaki R., Shibata M., Hasegawa Y., Satoh K., Tajima T., Takeuchi Y., Fujita T., Nakahara K., Yamashita T., Fukumoto S. | Increased circulatory level of biologically active full-length FGF-23 in patients with hypophosphatemic rickets/osteomalacia | 2002 | Journal of Clinical Endocrinology and Metabolism (Q1) | 579 | Dept. of Laboratory Medicine, , Tokyo, Japan |
| Liu S., Tang W., Zhou J., Stubbs J.R., Luo Q., Pi M., Quarles L.D. | Fibroblast growth factor 23 is a counter-regulatory phosphaturic hormone for vitamin D | 2006 | Journal of the American Society of Nephrology (Q1) | 542 | Department of Internal Medicine, 3901 Rainbow Boulevard, Kansas City, KS 66160, United States |
| Sitara D., Razzaque M.S., Hesse M., Yoganathan S., Taguchi T., Erben R.G., Jüppner H., Lanske B. | Homozygous ablation of fibroblast growth factor-23 results in hyperphosphatemia and impaired skeletogenesis, and reverses hypophosphatemia in Phex-deficient mice | 2004 | Matrix Biology (Q1) | 447 | Dept. of Oral and Devmtl. Biology, United States |
| Larsson T., Marsell R., Schipani E., Ohlsson C., Ljunggren Ö., Tenenhouse H.S., Jüppner H., Jonsson K.B. | Transgenic mice expressing fibroblast growth factor 23 under the control of the α1(I) collagen promoter exhibit growth retardation, osteomalacia, and disturbed phosphate homeostasis | 2004 | Endocrinology (Q1) | 439 | Department of Surgical Sciences, SE-751 85, Uppsala, Sweden |
| Liu S., Guo R., Simpson L.G., Xiao Z.-S., Burnham C.E., Quarles L.D. | Regulation of fibroblastic growth factor 23 expression but not degradation by PHEX | 2003 | Journal of Biological Chemistry (Q1) | 430 | Box 3036, Duke University Medical Center, Durham, NC 27710, United States |
